# Cardiac Arrest From Local Anesthetic Systemic Toxicity (LAST): A Rare Complication of Ultrasound-Guided Sternal Hematoma Block

**DOI:** 10.1016/j.acepjo.2025.100102

**Published:** 2025-03-20

**Authors:** Kate G. Radcliffe, Tessa Adler, Jordan Petersen, Kaitlen Howell, Arun Nagdev, David Martin

**Affiliations:** 1 School of Medicine, University of California, San Francisco, San Francisco, California, USA; 2 Department of Emergency Medicine, Highland Hospital - Alameda Health System, Oakland, California, USA

**Keywords:** ultrasound, nerve block, pain, sternal fracture, regional anesthesia

## Abstract

Local anesthetic systemic toxicity (LAST) is a rare but serious complication of ultrasound-guided nerve and hematoma blocks, affecting the cardiovascular and central nervous systems. Clinicians performing these procedures should be aware of the possibility of LAST, recognize the early signs and symptoms, and understand the current accepted treatment. We present a case of cardiovascular collapse caused by LAST following an ultrasound-guided sternal hematoma block and discuss potential systems-based improvements to enhance the management and prevention of this life-threatening emergency. Thoracic blocks may pose a higher risk of LAST because of the close proximity to the heart, and we recommend using lower doses of local anesthetic when performing these blocks.

## 1 Introduction

Local anesthetic systemic toxicity (LAST) is a rare but known dangerous complication of ultrasound-guided nerve and hematoma blocks, affecting the cardiovascular and central nervous system (CNS).^1^ The mechanism remains unclear but may be caused by local anesthetics binding to sodium channels in the CNS and heart. The incidence of LAST in ultrasound-guided nerve blocks is low, with estimated rates between 0.03% and 0.18%, mainly in perioperative settings.^2^, ^3^, ^4^ A review of 420 ultrasound-guided nerve blocks at a single emergency department (ED) revealed no cases of LAST over 1 year.^5^ No large studies have examined LAST in hematoma blocks, but there are documented cases of LAST following wrist hematoma blocks.^6^^,^^7^

In this report, we present a case of cardiovascular collapse secondary to LAST after an ultrasound-guided sternal hematoma block. Although rare, with the growing use of ultrasound-guided nerve blocks in the ED, clinicians performing these procedures must be prepared to recognize and treat LAST due to its potentially devastating effects.^8^ We present our experience managing this rare, life-threatening emergency and highlight systems-based interventions to improve care in similar cases.

## 2 Case Presentation

An 84-year-old woman weighing 50 kg was admitted to the trauma surgery service after sustaining a mildly displaced sternal body fracture in a motor vehicle accident. The patient’s medical history was notable for hypertension, heart failure with preserved ejection fraction, osteoporosis, and no prior history of seizure or arrhythmia, though she presented to the ED in atrial fibrillation. The patient’s pain remained poorly controlled after receiving 1000 mg of intravenous (IV) acetaminophen and 0.6 mg of IV hydromorphone. An ultrasound-guided sternal hematoma block was offered for analgesia. Before the block, the patient was in distress due to pain with an irregularly irregular heart rate of approximately 90 beats per minute and a blood pressure of 153/94 mm Hg.

**Figure 1:**
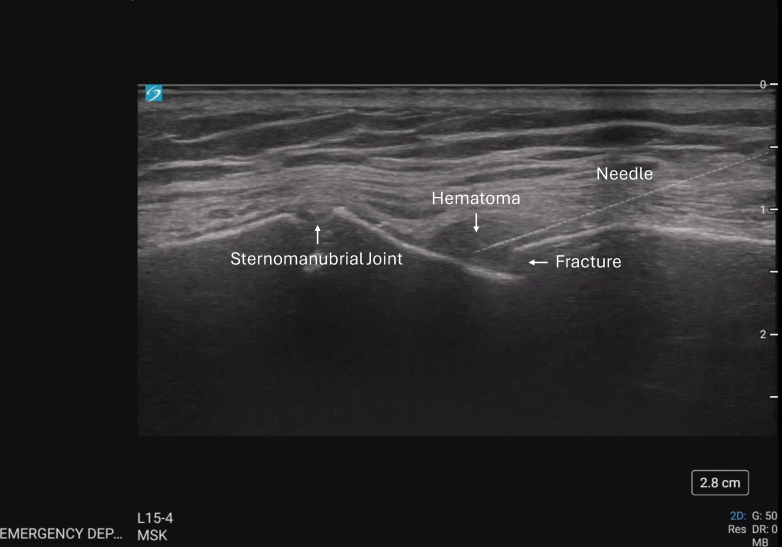
The block needle is positioned at the level of the fracture line, and anechoic fluid is visible surrounding the needle tip.

The sternal fracture and overlying hematoma were identified in a long-axis plane using a high-frequency linear transducer (SonoSite-Px). After cleaning the site, a skin wheal was made with a 25-gauge needle and 5 mL of lidocaine 1%. A 20-gauge block needle (Pajunk SonoBlock II) was advanced under ultrasound guidance until the needle tip abutted the fracture site (Fig 1); 20 mL of ropivacaine 0.5% (5 mg/mL) was injected in 3- to 5-mL aliquots. Aspiration was performed before injecting and intermittently throughout the procedure to ensure the lack of intravascular injection.

Approximately 60 seconds after completion of the block, the patient had a generalized tonic-clonic seizure. Her symptoms were immediately recognized as LAST. Intralipid was available at the bedside according to our protocol for all nerve and hematoma blocks. We administered an initial bolus of 75 mL IV intralipid (1.5 mL/kg). The seizure lasted approximately 1 to 2 minutes during which she was noted to be in atrial fibrillation with rapid ventricular response. Midazolam 2 mg IV was subsequently administered, terminating the seizure. Within minutes of the initial intralipid bolus, the patient became bradycardic, losing femoral pulses, and cardiopulmonary resuscitation (CPR) was initiated; 75 mL of intralipid was readministered.

During the initial pulse check, the patient was found to be in ventricular tachycardia and was defibrillated at 200 J. During the subsequent CPR pause, pulses were palpable, and cardiac compressions were halted. The patient remained in ventricular tachycardia and received 150 mg of IV amiodarone. Synchronized cardioversion was performed at 200 J, after which she was in atrial fibrillation with rapid ventricular response. Intralipid infusion of 0.5 mL/kg per minute continued during the medical resuscitation. Within 7 minutes of the return of spontaneous circulation, the patient became hypotensive, and low-dose norepinephrine infusion was initiated. The intralipid infusion was stopped when she no longer required norepinephrine. In total, we administered approximately 750 mL of intralipid in the first hour. She required an additional bolus of 75 mL (1.5 mL/kg) of intralipid for hypotension 1 hour after the infusion was stopped, which resolved the hypotension. She had no recurrent episodes of hemodynamic instability. She was extubated within 3 hours and was awake, alert, and following commands. She was discharged after 7 days, neurologically intact with no further cardiac events or seizure activity.

## 3 Discussion

Although rare, LAST is a potentially life-threatening complication to consider when performing ultrasound-guided nerve and hematoma blocks. Symptoms typically appear 1 to 5 minutes postinjection, although presentations up to 30 minutes after injection have also been reported.^2^^,^^9^^,^^10^ The American Society of Regional Anesthesia and Pain Medicine (ASRA) guidelines, therefore, advocate to observe patients for at least 30 minutes postinjection to monitor for signs of LAST. Patients with LAST may initially experience CNS excitation, including auditory changes, perioral numbness, metallic taste, and agitation, which can progress to seizures or CNS depression. CNS involvement has been reported to occur in 80% of patients with LAST, of whom two-thirds presented with seizures.^9^, ^10^, ^11^ If local anesthetic enters large central arteries, cardiac symptoms such as hypertension, tachycardia, and ventricular dysrhythmias may precede CNS symptoms, followed by cardiac depression, including bradycardia, conduction blocks, decreased contractility, and asystole. Up to one-third of patients with LAST present with signs of cardiac toxicity, including dysrhythmias, hemodynamic instability, and cardiac arrest.

The ASRA recommends a protocol for treating LAST based on anecdotal and animal data.^12^ This protocol involves using benzodiazepines for acute seizures and IV 20% lipid emulsion for cardiac dysfunction. The lipid emulsion should be administered in all cases of suspected LAST because it is believed to bind to the lipophilic local anesthetic, acting as a “lipid sink.” The Standard Advanced Cardiac Life Support protocols should be followed but with modifications: a reduced dose of epinephrine (1 μg/kg) and avoiding local anesthetic antiarrhythmics, beta-blockers, calcium channel blockers, and vasopressin due to their potential to worsen toxicity or cardiac depression.^10^^,^^13^ The recommended dosage is a 1.5 mL/kg bolus of 20% lipid emulsion, followed by a continuous infusion of 0.25 mL/kg per minute. If hemodynamic stability is not achieved, an additional 1.5 mL/kg bolus can be given, and the infusion rate can be increased to 0.5 mL/kg per minute. The maximum recommended dose for the initial administration is approximately 10 mL/kg over 30 minutes.

To prevent LAST, we recommend the following: keeping anesthetic doses within weight-based limits, ensuring continuous visualization of the needle tip during the procedure, repeatedly aspirating before injecting small volumes (3-5 mL) of anesthetic, and maintaining cardiac monitoring during the procedure and 30 minutes afterward. In addition, intralipid emulsion should be readily available to all clinicians performing ultrasound-guided nerve or hematoma blocks. At our institution, the intralipid is at the bedside during all ultrasound-guided nerve or hematoma blocks.

**Figure 2:**
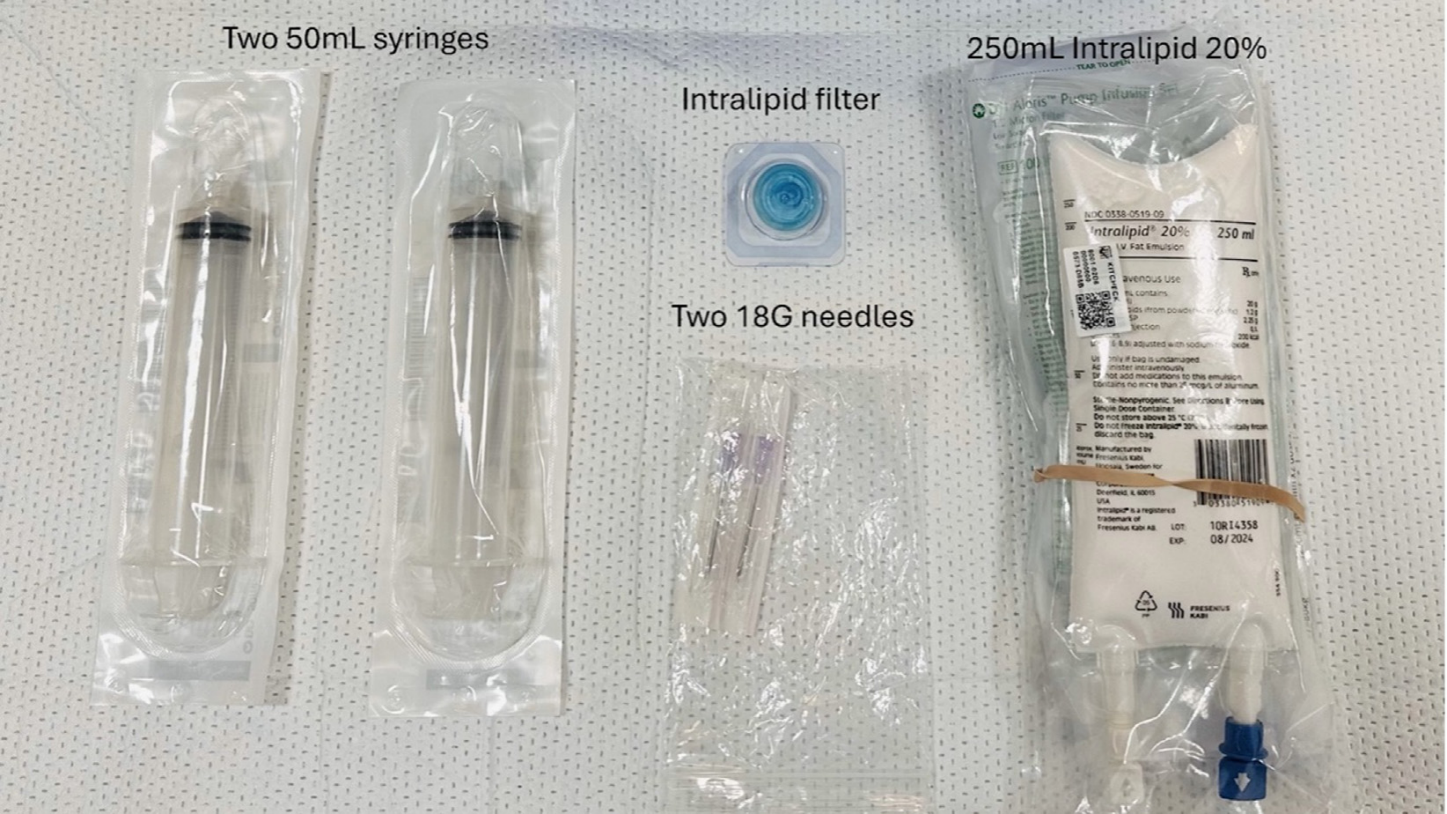
An intralipid kit that includes the necessary equipment for drawing up the initial IV intralipid bolus dose and administering the IV intralipid infusion.

Based on our experience, we recommend setting up an intralipid kit that includes two 50 mL syringes, a large bore needle (16-18G), an intralipid filter to draw up the initial bolus dose, and IV tubing with an intralipid filter for the IV infusion (Fig 2). The pharmacy guidelines recommend filtering of intralipid before administration to remove particulate matter and microorganisms.^14^ We, therefore, recommend using a syringe attached to a filter and needle to draw up the intralipid and then administering the intralipid in the syringe via IV access. EDs should institute policies to ensure intralipid is easily accessible following a nerve or hematoma block.

Data regarding the efficacy and safety of ultrasound-guided sternal hematoma and parasternal blocks for the treatment of sternal fractures are limited to case reports, which have not reported any adverse events.^15^, ^16^, ^17^, ^18^ There have been limited reports of adverse events following regional anesthesia of the anterior chest wall, which may be due in part to their emerging clinical use.^19^ A randomized controlled trial of continuous bilateral parasternal infusion of ropivacaine following cardiac surgery found that in 22% of patients, plasma levels of ropivacaine exceeded the safety threshold value; however, no patients reported symptoms consistent with LAST.^20^

Parasternal and sternal hematoma blocks may therefore carry a higher risk of LAST due to the chest wall’s vascularity and proximity to the heart, leading to rapid increases in plasma levels of local anesthetic. In our case, the dose of local anesthetic was approximately 85% of the maximum dose recommended (ropivacaine 3 mg/kg and lidocaine 5 mg/kg). In addition, patients sustaining sternal fractures are at an increased risk of cardiac contusions, making the heart more prone to preexcitation from local anesthetics. We, therefore, recommend using lower doses of local anesthetic when performing ultrasound-guided sternal hematoma and truncal area blocks.

## 4 Conclusion

Although rare, LAST is a potentially life-threatening complication during ultrasound-guided nerve and hematoma blocks. Clinicians must be aware of the risk of LAST, recognize its early signs and symptoms, and be familiar with treatment protocols. EDs should ensure that the intralipid is readily accessible following nerve or hematoma blocks. Thoracic ultrasound-guided blocks may carry a higher risk of LAST due to increased vascularity and proximity to the heart, leading to higher plasma levels of local anesthetic. Therefore, we recommend using lower doses of anesthetic when performing these blocks.

## Funding and Support

This research did not receive any specific grant from funding agencies in the public, commercial, or not-for-profit sectors.

## Conflict of Interest

The authors declare that they have no known competing financial interests or personal relationships that could have appeared to influence the work reported in this paper.
